# Inhibitional Effects of Metal Zn^2+^ on the Reproduction of *Aphis medicaginis* and Its Predation by *Harmonia axyridis*

**DOI:** 10.1371/journal.pone.0087639

**Published:** 2014-02-12

**Authors:** Guoqiang Xie, Jiaping Zou, Lina Zhao, Mengjing Wu, Shigui Wang, Fan Zhang, Bin Tang

**Affiliations:** 1 Hangzhou Key Laboratory of Animal Adaptation and Evolution, College of Life and Environmental Sciences, Hangzhou Normal University, Hangzhou, Zhejiang, China; 2 Institute of Plant and Environment Protection, Beijing Academy of Agriculture and Forestry Sciences, Beijing, China; Federal University of Viçosa, Brazil

## Abstract

**Background:**

Contamination, including metals, can disturb the reproductive processes of many organisms, including both prey and predatory insects. However, there is virtually no information on the effects of high level Zinc (Zn) pollution on aphids and ladybirds. The high concentrations of Zn^2+^ or Zn pollution inhibit reproduction in the phytophagous aphid, *Aphis medicaginis*, and the predatory ladybird *Harmonia axyridis* could provide important information.

**Results:**

It was observed in this study that Zn concentrations in *Vicia faba* (broad bean) seeds and seedlings in all Zn^2+^ treatments were significantly higher than that in the control group, and increased with increasing Zn^2+^ concentrations in the solution. The rate of reproduction in *A. medicaginis* declined significantly (p<0.05) over time in the five groups fed on broad bean seedlings treated with different concentrations of Zn^2+^ solution compared with the control group. These results showed that higher concentrations of Zn^2+^ significantly inhibited the reproductive capacity of *A. medicaginis*. We also cloned and identified a gene encoding vitellogenin (Vg) from *A. medicaginis*, which has an important role in vitellogenesis, and therefore, reproduction was affected by exposure to Zn^2+^. Expression of *AmVg* was reduced with increasing exposure to Zn^2+^ and also in the F_1_–F_3_ generations of aphids exposed to different Zn^2+^ concentrations. Predation by *H. axyridis* was also reduced in aphids exposed to high-levels of Zn^2+^. Similarly, ovipositioning by *H. axyridis* was also reduced.

**Conclusions:**

Our results suggest that Zn^2+^ can significantly affect the reproductive capacity of both *A. medicaginis* and its predator *H. axyridis*, the former through effects on the expression of *AmVg* and the latter through avoidance of aphids containing high levels of Zn^2+^.

## Introduction

Healthy soil is crucial to sustain the function of natural ecosystems. However, soil pollution has become a widespread environmental problem worldwide over the past few decades, particularly with metals originating from both natural and anthropogenic sources, such as chemical fertilizers, pesticides, and industrial sewage [1], [2]. Such pollution can also threaten human health [3], the environment and its accompanying biodiversity, as well has having detrimental effects on the development and reproduction of many species [4]. Metals have slow mobility, are not easily leached into water and cannot be degraded by microorganisms; thus, when metals in soil exceed the capacity of the environment, they affect plants directly, which often become enriched with such compounds [5], [6]. Insects are also significantly affected by metal pollution. Metals can be absorbed by insects through their spiracles, across their cuticles and through feeding; such absorption not only results in changes in the genetics of insects, but can also induce apoptosis and affect the viability and proliferation of cells, impacting insect growth and reproduction [7]. Metals can also accumulate in higher trophic-level organisms via the food chain [8].

Insects can accumulate large quantities of yolk proteins in eggs during the process of oogenesis. The protein precursor of yolk protein, vitellogenin (Vg), is synthesized in the fat body, released into the hemolymph and sequestered by developing oocytes to serve as a nutrient supply [9], [10], [11], [12]. However, metals are known to inhibit vitellogenesis, thus affecting the reproduction and physiology of affected individuals [13]. Various hypotheses have been proposed which suggest that metal stress reduces the expression of Vg mRNA, resulting in reduced deposition of Vn in eggs, and thus, a subsequent decline in fecundity and hatchability [14]–[20].

Excessive levels of metals also affect the normal functioning of insect cells and tissues; in particular, when divalent metal ions are transported across membranes into cells, they disturb both the extra- and intercellular ion balance, impacting the cell membrane polarity, pH stability, membrane permeability and the steady state of the intracellular environment [21], [22]. In addition, redox of metals can affect the function of ion channels and ion pumps in the plasma [23] and the insect immune defense system [24]. Insects are likely to consume substantial amounts of energy while attempting to overcome the effects of excessive metals; thus, normal physiological functions are likely to be seriously affected [14], such as: changes in generation time, body mass and reproductive capacity, reduction in fecundity, increased mortality, population decline and so on [25]–[27].

The transfer and accumulation of metals along the food chain accelerates the deterioration of the ecological environment and influences the metabolism and development of organisms in various ecosystems [28]. It also has a potential impact on the development and metabolism of phloem-feeding insects [2]. The expression of the heat shock protein (Hsp) 70 can act as a marker of cellular damage sustained by insects in polluted habitats and by exposure to Zinc (Zn) during diapause [19], [20], [29]. Tributyltin (TBT) and cadmium (Cd) tested on the freshwater arthropod *Chironomus riparius* (Diptera), resulted in inhibition of oviposition [30]. In addition, Cd inhibited vitellogenesis in the milkweed bug *Oncopeltus fasciatus* (Heteroptera: Lygaeidae) [31].

China is one of the largest global producers and consumers of metals such as lead (Pb) and zinc (Zn) [32], with a density greater than 5 g/cm^3^ [33]. It was found that the average concentration of Zn in China was 498 mg/kg in paddy soil [34] and 166.9 mg/kg in soil where vegetables were growing [35]. In addition, it was reported that the concentrations of Zn in agricultural and non-agricultural soil were in the range of 65.7 to 766 mg/kg and 34.7 to 193 mg/kg in the Pearl River of Guangdong China, respectively [36]. Zn^2+^ is a highly toxic metal which is widely dispersed in the environment, mainly as a result of animal activities, and affects selective neuronal death after transient global cerebral ischemia [37]. Zn is potentially toxic to organisms at concentrations which significantly exceeds physiological limits, and can affect insect reproduction [19], [38], [39]. Plant phytophagous insects and predators are an important part of the food chain in nature. Toxic metals can be absorbed by plants and then transferred to phytophagous and predator insects [40].

It has been reported that certain concentrations of metals can have a potential impact on the survival and reproduction of both prey and predatory insects [41]. Aphids are very important and widespread pests and the ladybird is a good predator for controlling the density of aphids in agroecological systems. However, there is virtually no information on the effects of high level Zn pollution on aphids and ladybirds. Because Zn^2+^ can be transferred and accumulated in the food chain, and can affect insect development and fecundity, we hypothesized that high concentrations of Zn^2+^ or Zn pollution may inhibit reproduction in the phytophagous aphid, *Aphis medicaginis*, and the predatory ladybird *Harmonia axyridis*. Therefore, we used aphids and ladybirds to test our hypothesis in this study. Here, we report the outcomes of the experiments designed to test our hypothesis.

## Materials and Methods

### Test insects

Colonies of *H. axyridis* and *A. medicaginis* were maintained in our laboratory over a 3-year period. The colonies were reared at 25±1°C under an L14: D10 photoperiod and 50%–75% relative humidity.

### Treatment design and Zn accumulation in *Vicia faba* seeds and seedlings

Zn chloride was diluted with water to a Zn mass ratio of 0 (only tap water was added and acted as the control group: CK), 50, 75, 100, 125 and 150 mg/kg. *Vicia faba* (broad bean) seeds were water-soaked for 12 hours with the different concentrations of Zn^2+^-containing solutions, then planted in soil and watered with the corresponding solutions.

The seeds and seedlings which were water-soaked and watered using 0, 50, 75, 100, 125 and 150 mg/kg were vacuum dried at 60°C for 24 h in Pyrex test tubes. Five hundred milligrams of samples (dry weight) were digested in 10 ml of boiling nitric acid (65%) and 1 ml concentrated perchloric acid (BAKER ANALYZED reagent; Baker, Deventer, Holland) [39], [40]. When the fume was white and the solution was completely clear, the samples were cooled to room temperature. After filtrating using filter paper, the clear solution was transferred to a volumetric flask which was then filled to 50 ml with deionized water. Zn concentrations were estimated using an inductively coupled plasma-atomic emission spectrometer (ICP-AES, Thermo Jarrell Ash Company, USA). Concentrated nitric acid and perchloric acid were used as the blank control. Zn concentrations in seeds and seedlings were calculated as follows: Concentration of Zn = (C×50)/500 mg, where C is the Zn concentration detected by ICP-AES.

### The influence of different concentrations of Zn^2+^ on the reproduction of *A. medicaginis*

In the *A. medicaginis* reproduction experiment, similar diluted Zn solutions were used and *Vicia faba* seeds were planted in the same way. *Vicia faba* seeds were watered with the corresponding solutions as described previously. In excess of 30 seeds were planted for each Zn^2+^ treatment. Three mother *A. medicaginis* were removed from the laboratory colony and placed on each broad bean seedlings when they were 10 cm high. Their reproductive rate was determined over a 7-day period.

### RNA extraction and first-stand cDNA synthesis

Five to ten fresh aphids, including F1 to F3 generations, from each Zn^2+^ treatment were collected in an Eppendorf tube which was repeated 3 times while the aphids were breeding. Total RNA from these aphids was extracted using Trizol reagent, and its integrity determined using agarose gel electrophoresis. Quantification of the concentration of RNA was performed using an ultramicro nucleic acid protein tester. Then, 1 µg of total RNA was taken as a template to synthesize first-stand cDNA using an RT-kit.

### Cloning and analysis of the *Aphis medicaginis Vg* gene

Eight specific primers (Table 1) were designed from open reading frames (ORFs) based on known *Vg* genes of *Acyrthosiphon pisum*. Polymerase Chain Reaction (PCR) amplification was carried out using the cDNA of *A. medicaginis* as the template. In total, the PCR system was 25 µl, comprising cDNA template 1 µl, 10×Taq Buffer 2.5 µl, dNTP mixture 2 µl, forward and reverse primers each 1 µl, Taq enzyme 0.2 µl, and double distilled water up to 25 µl. The PCR conditions were as follows: pre-denaturation at 94°C for 10 min, 31 cycles of 30 s at 94°C, 30 s at 48°C, 2 min 50 s at 72°C, and then 72°C for 10 min. After the reaction, the products were subjected to agarose gel electrophoresis. The DNA bands corresponding to the expected size were excised from the agarose gel and purified using a DNA gel extraction kit. These PCR products were cloned into the T vector and sent for sequencing. The resulting protein sequence was compared to those in the NCBI library, and the results showed that the sequence was that of the *Vg* gene of *A. medicaginis* (*AmVg*).

**Table 1:** PCR primers used in this study.

PCR fragment	Primer name	Nucleotide sequences (5′-3′)
Specific primers	ApmedVg-FA	ATG CAC GAC AGA CTG GGC TTT ATA GTC
	ApmedVg-FB	GTC GCC GTC GTA TCG TGT TAT CTG
	ApmedVg-FC	CGT GTG GTA AAG GAA TGT GCT C
	ApmedVg-FD	CAC TTC ACA CAG TCG ACA AAT GC
	ApmedVg-RA	GAG CAC ATT CCT TTA CCA CAC G
	ApmedVg-RB	GCA TTT GTC GAC TGT GTG AAG TG
	ApmedVg-RC	TCC CTC ATC TTG GCA GTC G
	ApmedVg-RD	TCA CTG ATA GTT TTT GTT ACT AAT CTG
Degenerate primers	Actin-DPF1	GGT GTM ATG GTH GGH ATG GG
	Actin-DPF2	GGT ATY CTY ACC YTG AAR TAC C
	Actin-DPF3	CCA ACT GGG AYG AYA TGG AG
	Actin-DPR1	TGG AAV AGV GMY TCG GG
	Actin-DPR2	CGA TDC CVG GRT ACA TGG TGG
	Actin-DPR3	GAG ATC CAC ATC TGY TGG A
Real-time qPCR primers	ApmedVg-qF	CCA TTC CAG GCA TTA GCA G
	ApmedVg-qR	CCC ATT GTA GGA TCT TCA TAG TG
	Actin-qF	GAC CGA AGC TCC ATT GAA CCC
	Actin-qR	CCA GAG TCC AAA ACG ATA CCA GTG

### Sequence and data analysis

Sequence and system analysis utilized the online analysis tools DNAstar, Compute pI/Mw and ClustalW (http://expasy.org/tools/#translate, NetNGlyc 1.0 Server: http://www.cbs.dtu.dk/services/NetNGlyc/; TMHMM Server v. 2.0: http://www.cbs.dtu.dk/services/TMHMM-2.0/; ClustalW: http://www.ebi.ac.uk/Tools/clustalw2/index.html, and SignalP 3.0 Server: http://www.cbs.dtu.dk/services/SignalP/) Other data analysis was carried out using Statistica 6.0. The phylogenetic tree was constructed using Mega 5.05 and *Oscheius tipulae* (OSU35449) and *Haemaphysalis longicornis* (AB359899) as the outgroups.

### *E*xpression of *AmVg* in response to different concentrations of Zn^2+^

Quantitative real-time PCR (qRT-PCR) was used to determine the relative expression level of *AmVg* gene in response to different concentrations of Zn^2+^. Internal reference primers and probes (Table 1) were designed based on the *beta-actin* gene of *A. medicaginis* and the conserved region of its *Vg* gene. Total RNA in *A. medicaginis* was extracted (see above), and its purity and concentration determined using gel electrophoresis and ultramicro nucleic acid protein testers. One microliter of total RNA was used for first-stand cDNA reverse transcription. The qRT-PCR system was 20 µl in total, comprising SYBR mix 10 µl, DEPC-treated water 7 µl, forward and reverse primer each 1 µl, and template cDNA 1 µl. qRT-PCR was carried out in a C1000™Thermal Cycler (BioRad) under the following conditions: pre-denaturation at 95°C for 3 min, 39 cycles of 10 s at 95°C, 30 s at 57°C, 30 s at 65°C, and then the fluorescence signal was collected at 65°C. The data were analyzed with the program supplied with the quantitative PCR instrument.

### The influence of different concentrations of Zn^2+^ on predation by *Harmonia axyridis*

The reproductive rates of *A. medicaginis* fed on broad bean seedlings treated with 50, 75 or 100 mg/kg Zn^2+^ were significantly different, and the reproductive rates in the groups treated with 125 or 150 mg/kg Zn^2+^ were not significantly different during the seven day period (p>0.05). Therefore, the *Vicia faba* seeds were first watered, planted in soil and then watered with different Zn^2+^-containing solutions (100 and 150 mg/kg) in subsequent experiments. Aphids were removed from the seedlings watered with different levels of Zn^2+^ solution.

Business fly tubes were marked as follows (where CK is the control group): CK-20, CK-60, CK-100, CK-140, 100-20, 100-60, 100-100, 100-140, 150-20, 150-60, 150-100 and 150-140. Each experiment was repeated 30 times. *A. medicaginis* were selected from the *A. medicaginis* of the control group (CK) that were smaller than mother *A. medicaginis* (to avoid them multiplying in the tubes) and put into the plastic tubes. Twenty *A. medicaginis* were put in the tube marked 20, 60 put into the business fly tube marked 60, and so on for the tubes marked 100 and 140. The *H. axyridis* adults used in the experiment had been denied food for 24 h. Business fly tubes marked 1–15 were loaded with a female *H. axyridis*, whereas those marked with 16–30 were loaded with a male *H. axyridis*. The number of aphids remaining was used to determine the predation rate and, therefore, the aphid survival rate of *A. medicaginis* after 24 h in the artificial climate chamber.

### The influence of different concentrations of Zn2+ on the ovipositioning rate of *Harmonia axyridis*

Business fly tubes were marked as follows: CK-140, 100-140 and 150-140, and the experiments were replicated thirty times. Then, 140 *A. medicaginis* were selected from each of the broad bean populations of the control group (CK), and those watered with 100 mg/kg or 150 mg/kg Zn^2+^ solutions. A single mating *H. axyridis* female adult, which had previously been deprived of food for 24 h, was placed in each of the plastic tubes and left for a further 24 h. The number of *H. axyridis* eggs laid in each tube was then recorded.

### Statistical Analysis

Results are expressed as the mean ± standard error (SE) of different independent replicates (*n*≥3). The statistical significance of differences in reproduction of *A. medicaginis* and the relative expression levels of *AmVg* were determined by one-way analysis of variance (ANOVA) and analyzed by Tukey's test. Comparisons of different conditions were made with a two-way (ANOVA) followed by Tukey's test. .The significance level was set at α = 0.05.

## Results

### Zn accumulation in *Vicia faba* seeds and seedlings

**Figure 1:**
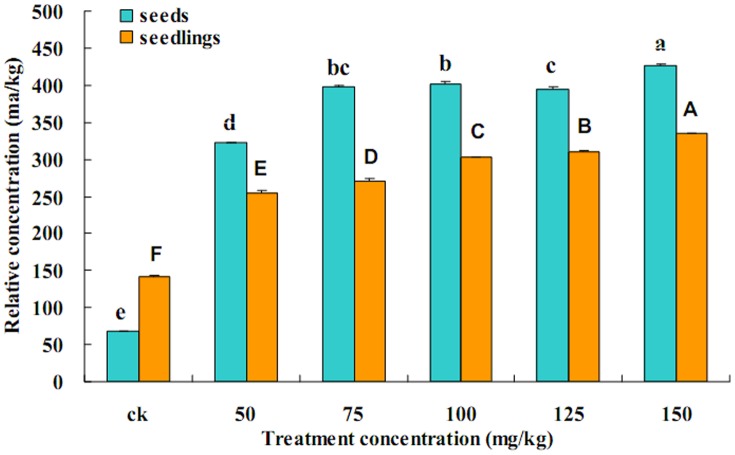
Zn accumulation in *Vicia faba* seeds and seedlings. *Vicia faba* (broad bean) seeds were water-soaked for 12 h with 0 (only tap water was added as the control: CK), 50, 75, 100, 125 and 150 mg/kg Zn^2+^-containing solutions, planted in soil and then watered with the corresponding solutions. The Zn concentrations in the seeds water-soaked for 12 h and in five-day seedlings were determined. Each treatment was repeated 3 times. (Tukey's test, α = 0.05, a>b>c>d>e>f or A>B>C>D>E>F)

The Zn concentrations in seed and seedlings in all treatments were significantly higher than the CK, and the concentration in the seeds and seedlings increased with increasing Zn concentrations in the solution (Fig. 1). The concentrations of Zn were 67.90 mg/kg and 322.20 mg/kg in the seeds of the CK and water-soaked 50 mg/kg Zn^2+^ solutions, respectively. The concentrations of Zn were high and reached 426.61 mg/kg following exposure to increased concentration of Zn^2+^ in the solutions. The Zn concentration in seedlings was greater higher than that in seeds and reached 141.23 mg/kg. The Zn concentrations in seedlings reached 335.17 mg/kg. These differences were significant as the concentration of Zn^2+^ in solutions increased from 50 mg/kg to 150 mg/kg.

### Effects of different Zn2+ concentrations on the reproductive rate of *A. medicaginis*

**Figure 2:**
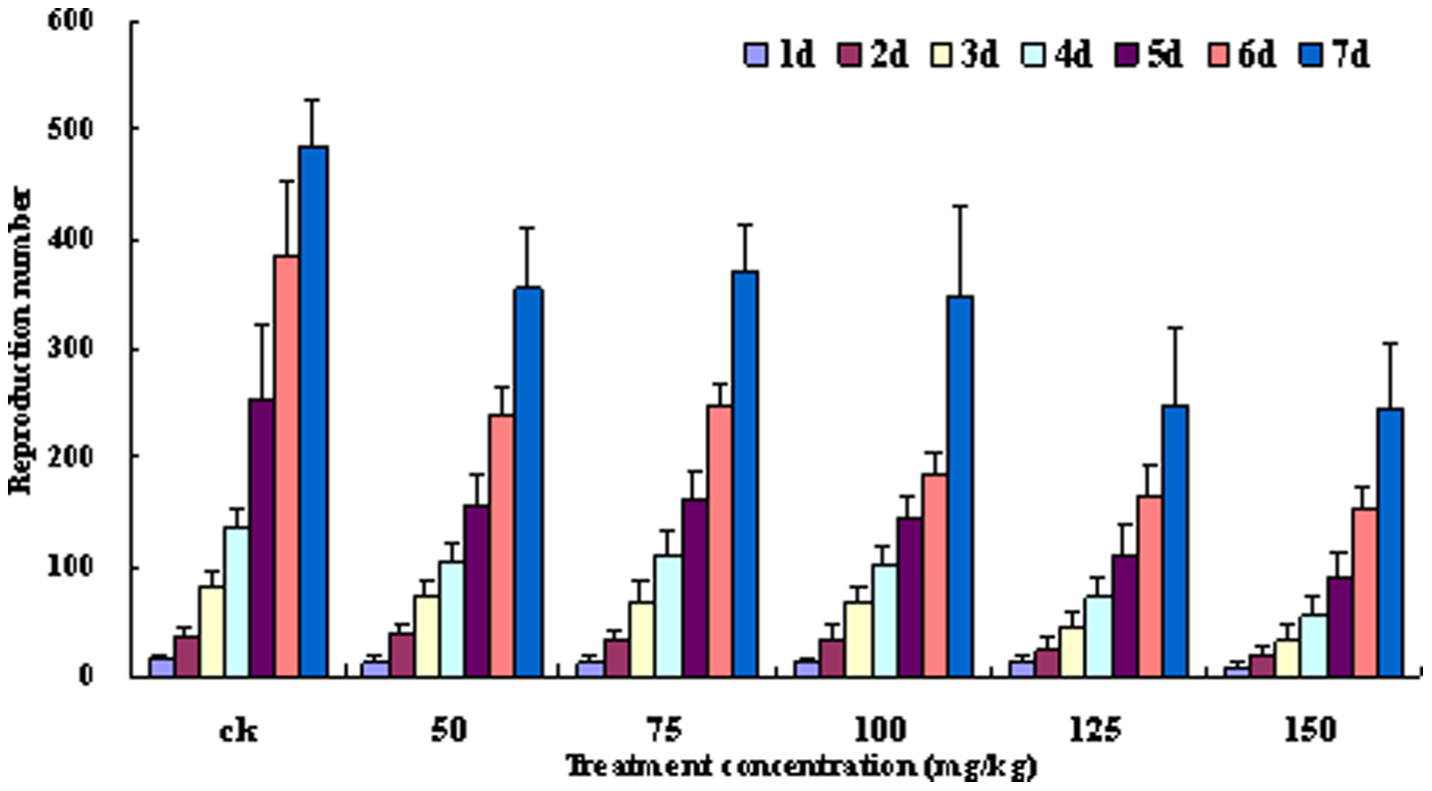
Reproduction of *Aphis medicaginis* at different Zn^2+^ concentrations. The reproductive capacity of *Aphis medicaginis* on seedlings watered with different Zn^2+^ solutions (50, 75, 100, 125 or 150 mg/kg) or with tap water (control: CK). Each treatment was repeated 30 times, and the numbers of *A. medicaginis* were recorded at intervals of 24 h. (Tukey's test, α = 0.05, a>b>c)

The results showed that the reproductive rate of *A. medicaginis* declined significantly (p<0.05) over time in the five groups fed on broad bean seedlings treated with different concentrations of Zn^2+^ compared with the control group (Fig. 2). The higher concentration of Zn^2+^, the lower the reproductive rate. Although those *A. medicaginis* fed on broad bean seedlings treated with 50, 75 or 100 mg/kg Zn^2+^ differed in their reproductive rates, the differences among them were not significant (p>0.05). Similarly, the reproductive rates of the groups where the seedlings had been treated with 125 or 150 mg/kg Zn^2+^ were not significantly different (p>0.05). Thus, the presence of Zn^2+^ in the soil is likely to impact negatively on the reproduction of *A. medicaginis*, with the impact increasing with increasing Zn^2+^ concentration.

### The cloning and analysis of the cDNA of *AmVg*

Given that the *Vg* gene of *A. medicaginis* has high homology with that of *Acyrthosiphon pisum*, the latter was used to design specific primers (Table 1). Following cloning, two bands of approximately 1500 bp were spliced and the protein sequences translated. *AmVg* was identified by comparing with records within the NCBI, and was registered as JX974432.

**Figure 3:**
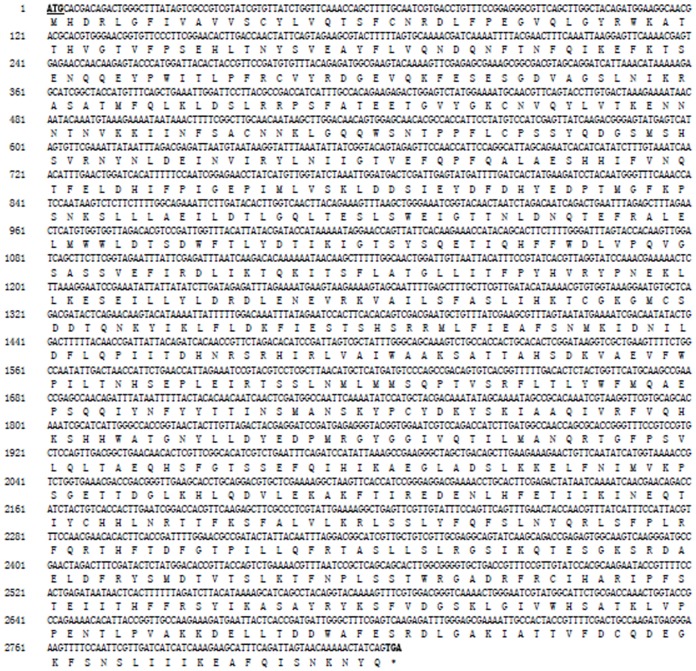
The sequence analysis of *Aphis medicaginis* Vg cDNA. Deduced nucleotide and amino acid sequences of the *Vg* gene of *Aphis medicaginis*. Both initiation and termination codons are indicated by bold and italics, the termination codon before the first Met is also indicated by bold and italic.

The results showed that the ORF of the *AmVg* gene was 2826 bp in length, translating 941 amino acids. Its isoelectric point was 6.44, and the predicted molecular weight of the protein was 108.68 kDa (Fig. 3). A potential transmembrane structure was found at 5 aa-22 aa based on analysis with TMpred; amino acids 1–22 were analyzed as the signal peptide with the online Signalp 3.0 Server. In addition, *AmVg* had nine N-glycosylation sites based on analysis with NetNGlyc, at amino acids 54, 68, 160, 169, 239, 282, 312, 525 and 727.

### Evolutionary analysis of the *AmVg* gene and those of other insects

**Figure 4:**
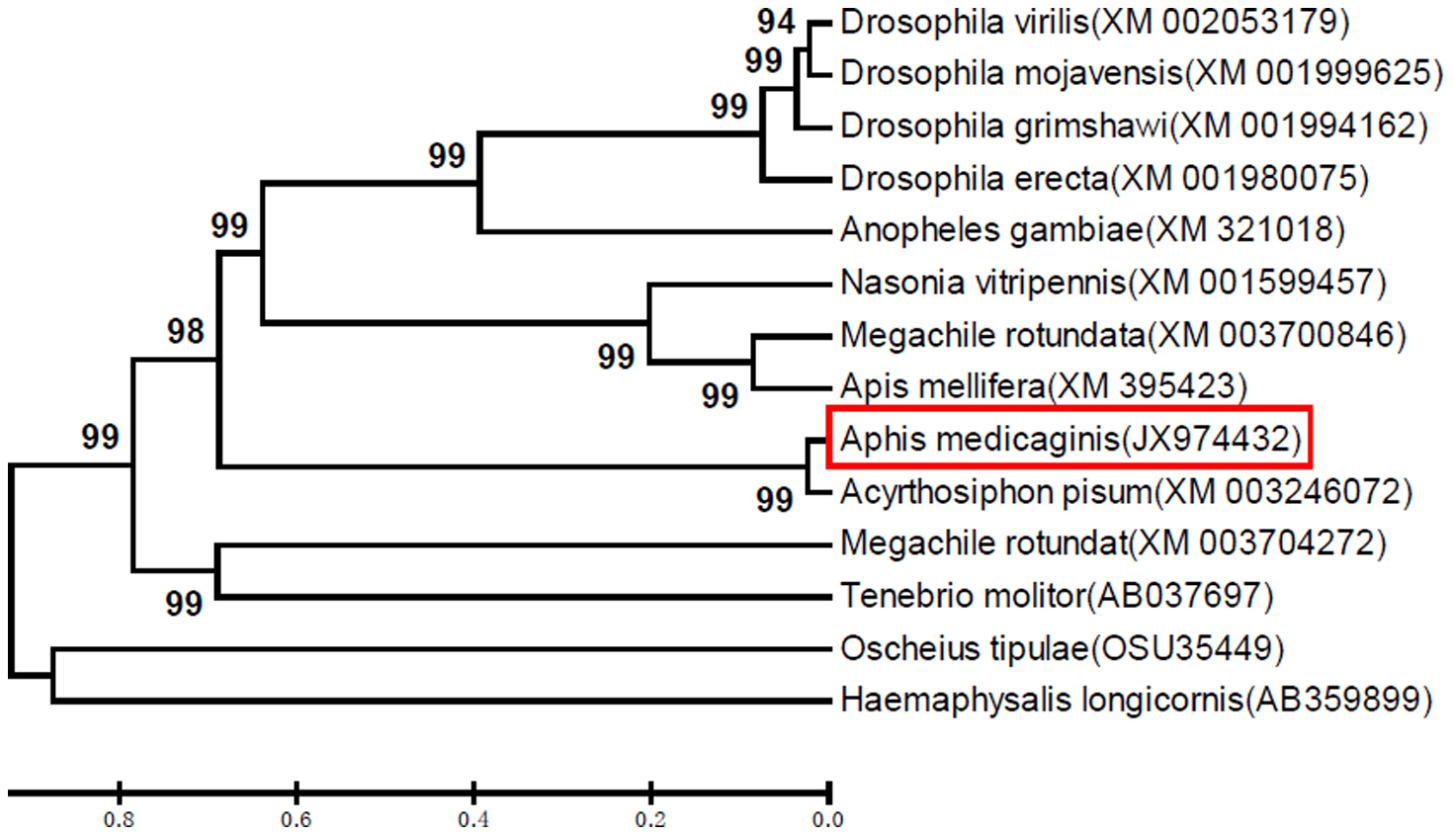
Phylogenetic analysis of the insect Vg gene amino acid sequences. Phylogenetic analysis of *Vg* genes from *Aphis medicaginis* and other species. The phylogenetic tree was constructed based on the amino acid sequences of known insect *Vg* genes. Full-length amino acid sequences were aligned using the Mega 5.05. A bootstrap analysis was carried out, and the robustness of each cluster was verified with 1000 replicates. Values at the cluster branches indicate the results of the bootstrap analysis. Vg genes were from *Acyrthosiphon pisum* (XM_003246072), *Megachile rotundata* (XM_003700846), *Apis mellifera* (XM_395423), *Nasonia vitripennis* (XM_001599457), *Drosophila virilis* (XM_002053179), *Drosophila mojavensis* (XM_001999625), *Anopheles gambiae* (XM_321018), *Drosophila grimshawi* (XM_001994162), *Drosophila erecta* (XM_001980075), *Megachile rotundata* (XM_003704272) and *Tenebrio molitor* (AB037697). The *Oscheius tipulae* (OSU35449) and *Haemaphysalis longicornis* (AB359899) genes were used as the outgroups.

The deduced amino acid sequence of *AmVg* was aligned with Vg genes from other species, and the sequences plus their accession numbers are listed in Fig. 4. Comparison of the protein sequences encoded by *Vg* genes from these insects with that encoded by *AmVg* showed the sequences are not highly conserved, with homologies ranging from 22% to 95%. *AmVg* was most similar (95% homology) to *Vg* from *Acyrthosiphon pisum* and least similar to that of the tick *Haemaphysalis longicornis* and the ant *Camponotus floridanus* (both 4%). The other homologies were as follows: 12% (*Oscheius tipulae*); 20% (*Tenebrio molitor*); 21% (*Megachile rotundata* and *Drosophila erecta*); 22% (*Drosophila mojavensis*, *Anopheles gambiae* and *Drosophila grimshawi*); *23%* (*D. virilis*); and 28% (*Apis mellifera* and *Nasonia vitripennis*).

### The expression of *AmVg* under different concentrations of Zn2+

**Figure 5:**
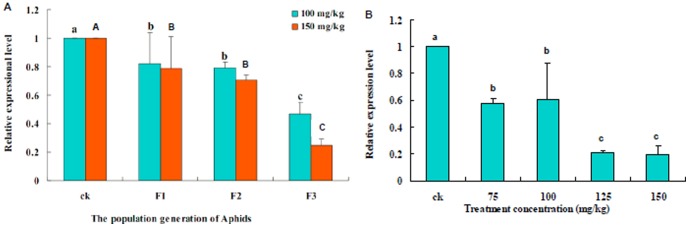
An analysis of *AmVg* transcripts in aphids from F1 to F3 generation by quantitative real-time PCR. **A**: The expression level of the *Vg* gene under different concentrations of Zn^2+^ in the F_1_, F_2_ and F_3_ generations of *Aphis medicaginis*. The developmental expression of *AmVg* was analyzed by qRT-PCR from aphids on broad bean seedlings watered with Zn^2+^ solutions of 0, 100 or 150 mg/kg. **B**: The expression of *AmVg* was analyzed by qRT-PCR from the F_4_ generation of aphids exposed to broad bean seedlings watered with Zn^2+^ solutions of 0, 75, 100, 125 and 150 mg/kg. (Tukey's test, α = 0.05, a>b>c or A>B>C)

The relative expression of *AmVg* in aphids exposed to different concentrations of Zn^2+^ was determined through qRT-CR. Fig. 5A presents the expression level of *AmVg* in mother *A. medicaginis* and then in her F_1_, F_2_ and F_3_ generations of aphids exposed to different levels of Zn^2+^ and a control group (CK). It shows that the expression of *AmVg* in each of the mother aphids exposed to Zn^2+^ decreased compared with the mother aphids from the control group. There was almost no difference in the expression of *AmVg* in the Zn^2+^-treated groups (100 and 150 mg/kg) in the F_1_ generation, but the difference increased with the increasing number of generations, with the expression being significantly different in the control versus the 100 and 150 mg/kg treated F_3_ aphids. Thus, exposure of aphids to Zn^2+^ over four generations had two main effects on the expression of *AmVg*: the first was that its expression decreased as the Zn^2+^ concentration increased (Fig. 5A); the second effect was that its expression between the two treated groups (100 and 150 mg/kg) reduced in the later generations. This result was consistent with the variation in reproductive capacity of the aphids.

The expression of *AmVg* was also investigated in aphids from the F_4_ generation and was also found to be reduced, supporting the trend recorded from the first three generations (Fig. 5B). These results showed that increasing Zn^2+^ concentration significantly (p<0.05) inhibited the reproduction of *A. medicaginis* in the aphids from the control group compared with those from the 75, 100, 125 and 150 mg/kg Zn^2+^ groups (no significant effect was recorded between the treatment groups themselves).

### Effect of Zn^2+^ treatment on A. medicaginis predation by *H. axyridis*

**Figure 6:**
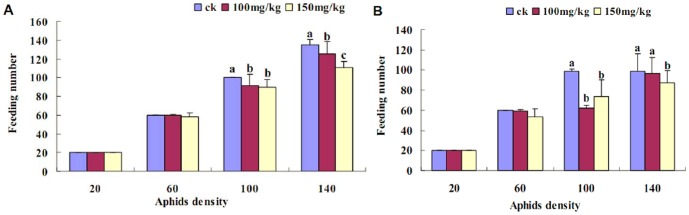
Feeding of *Harmonia axyridis* on aphids at different densities. *Aphis medicaginis* was predated by *Harmonia axyridis*. Different densities of *A. medicaginis* adults (20, 60, 100 or 140 per tube) were exposed to either female (A) or male (B) *H. axyridis* adults that had been denied food for 24 h. One *H. axyridis* adult was put in each tube and allowed to feed on the aphids for 24 h. The number of aphids remaining was used to determine the predation rate and, therefore, the aphid survival rate. (Tukey's test, α = 0.05, a>b>c)

In this experiment, the predation rate of *H. axyridis* was determined among four different densities of *A. medicaginis* (20, 60, 100 and 140 per tube). The female and male *H. axyridis* preyed on all *A. medicaginis* treated with different concentrations of Zn^2+^ because of the lack of an alternative food supply (Fig. 6). When the density of *A. medicaginis* exceeded the predatory amount of *H. axyridis*, the predatory amount of *H. axyridis* in each group decreased as Zn^2+^ concentration increased, for the same density of *A. medicaginis*. For example, in the 140-density group, *H. axyridis* males preyed on average on 98.8 *A. medicaginis* in the control group, 96.6 in the 100 mg/kg group, and 87 in 150 mg/kg group, whereas *H. axyridis* females preyed on average on 135 *A. medicaginis* in the control group, 125.3 in the 100 mg/kg group, and 110.3 in the 150 mg/kg group. Thus, we suggest that *H. axyridis* selectively preyed on those *A. medicaginis* that had not been exposed to Zn^2+^.

### Spawning rate of *H. axyridis*

**Figure 7:**
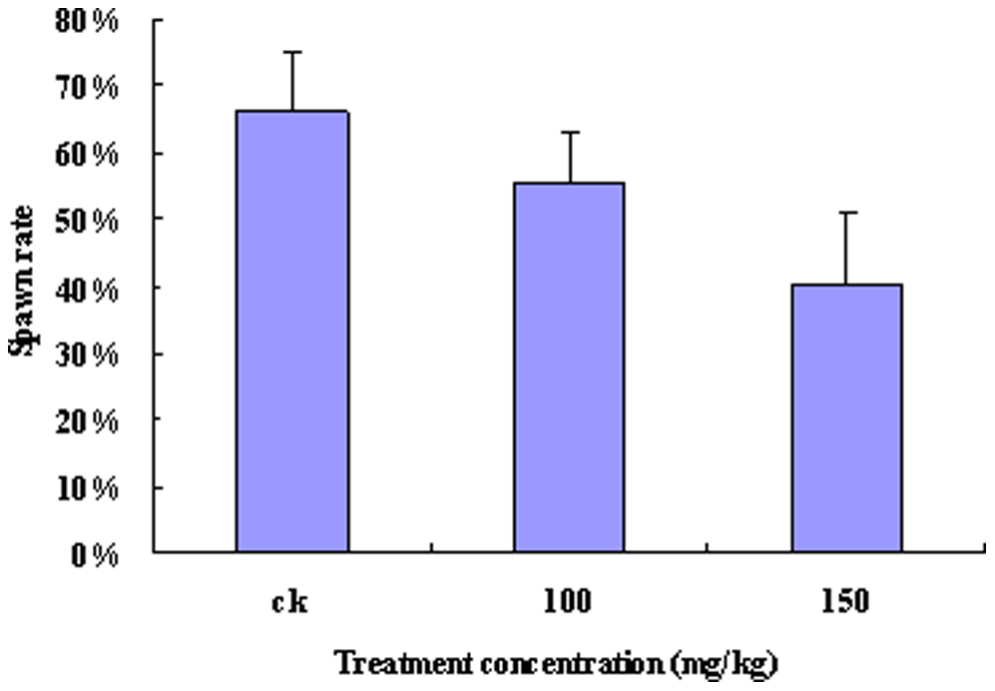
Rate of offspring in *Harmonia axyridis*. Female *H. axyridis* adults were divided into three groups and starved for 24 h before the experiment. One *H. axyridis* female adult was placed in each tube containing 140 aphids (from the broad beans watered with Zn^2+^ solutions of 0, 100 or 150 mg/kg) and allowed to feed for 24 h. The ovipositioning rate of each beetle was then determined. (Tukey's test, α = 0.05, a>b>c)

The ovipositioning rate of *H. axyridis* differed (p<0.05) depending on the Zn^2+^ concentration to which its aphid prey had been exposed. For example, the ovipositioning rate in *H. axyridis* fed on aphids from the control group reached 66%, whereas that of the *H. axyridis* fed aphids from the treatment groups (100 and 150 mg/kg Zn^2+^) were 55% and 40%, respectively (Fig. 7). These results showed that the reproductive capacity of *H. axyridis* can be influenced when feeding on *A. medicaginis* exposed to high levels of Zn^2+^.

## Discussion

Vg was first discovered by Telfer *et al.* (1954), who identified a female special protein (FSP) in *Hyalophora cecropia* [42]. In 1969, Pan renamed it vitellogenin [43]. Vg is a macromolecule phospholipid glycoprotein containing Ca and Zn ligands [44], [45], [46], and has many similar characteristics in vertebrates and invertebrates. It has been cloned from many organisms, including *Athalia rosae* [47], *Xenopus iaevis* [48], *Pteromalus puparum* [49], lobster [50], *Tigriopus japonicus* [51], and *Nilaparvata lugens* [52]. Our results showed that *A. medicaginis* Vg mRNA was first cloned and the homologies among the Vg protein sequence in different insects were found to be low and similar to those in insects in the same family or order (Fig. 4).

Reproductive and developmental disorders have frequently been associated with metal exposure in different organisms, including insects [30], [53]. For example, solid wastes from tanneries can have detrimental effects on the development and reproduction of *Drosophila melanogaster* [29]. The potential for the uptake of metals by aphids was demonstrated by Crawford *et al.* (1995), who observed the uptake and accumulation of Cd in the black bean aphid, *Aphis fabae*, indicating a potential transfer route of Cd from wheat to aphids [44]. Cd and Zn can also undergo bioaccumulation in the grain aphid, *Sitobion avenae* [54], [55], [56] and ladybird [56]. It can be seen from the results shown in Fig. 1 and Fig. 2 that the aphid reproductive rate decreased with increased Zn^2+^ content in *Vicia faba* seedlings. This showed that aphid vitellogenin protein synthesis may be affected. These findings also showed that a high concentration of Zn inhibits the reproduction of *A. medicaginis* (Fig. 2). In a previous study, Zn, Manganese (Mn) and Copper (Cu) were detected in the mandibles and ovipositors of gall-inducing wasps [57]. Although Zn is an essential microelement for animal nutrition [58], it impacts negatively on organismal growth and development if its concentration in an organism exceeds the physiological limits, especially in insects. For example, levels of Zn in combination with female aging were shown to have important effects on nymphal life history in a grasshopper species from polluted sites [19]. In addition, Zn is also known to be a teratogen [59], [60], [61], [62], [63], [64], to cause a decline in body mass [65], to cause a decrease in spawning rate [19], [38], [66], to reduce life-cycle length [18], and to even inhibit feeding [67], [68], [69].

As previously mentioned, TBT and Cd both inhibited ovipositioning by *C. riparius* [30]. Cd can also inhibit vitellogenesis in *Oncopeltus fasciatus* females (Heteroptera: Lygaeidae) [31]. Reproduction of *A. medicaginis* in the CK was higher than that in the aphids exposed to Zn^2+^-containing solutions (Fig. 2). In addition, the Zn concentration in seedlings was greater than that in seeds and reached 141.23 mg/kg in the CK (Fig. 1), which showed that soil and tap water may contain a certain concentration of Zn metal. From the results shown in Fig. 1 and Fig. 6, with an increasing Zn^2+^ concentration in *Vicia faba* seedlings, there was a concomitant decrease in the reproduction of *A. medicaginis*, in predation of *A. medicaginis* by *H. axyridis*, and also in ovipositioning by *H. axyridis*. An investigation on grasshoppers and *Spodoptera litura* showed that the number of eggs laid by aging females decreased gradually in insects exposed to Zn (19, 39), and springtails (*P. minuta*) suffered a reduction in adult survival and reproduction at high concentrations of Zn (38). In this study, aphid reproduction and ovipositioning were inhibited when the Zn concentration in *Vicia faba* seedlings was greater than 250 mg/kg.

Absorption of metals from the environment by insects (e.g. across the cuticle, via spiracles or ingestion) can result in changes to the cellular ultrastructure and genetics of the insect; for example, expression of Vg mRNA was found to be downregulated and the accumulation of Vn in eggs reduced in response to metal pollution [18]. Shu also showed that high levels of Zn reduced the expression of *Vg* in *Spodoptera litura* and negatively affected its reproduction [39]. Gene expression profiles of insect can be used to distinguish different responses to toxins such as metals. Our results showed that, with increasing Zn^2+^ concentration in *Vicia faba* seedlings and increasing generations of *A. medicaginis*, the expression of *AmVg* was gradually reduced, confirming the effect of high-level Zn on the expression of this gene in insects (Fig. 5A and Fig. 5B). De Schamphelaere suggested that the decline in reproduction caused by metals might be related to the direct effects of metals on reproductive processes, such as Vg synthesis [14]. Vg in insects is synthesized in the fat body, transported to the oocyte through the hemolymph, taken up by the oocyte and accumulates in eggs in the form of Vn. It is known that metals accumulate mainly in the fat body and eggs of insects [70], which indicate that metals can been accumulated via the food chain.

There have been several validation studies on the suitability of reference genes in different invertebrate species, including beta-actin, armadillo, elongation factor 1 alpha, 18SrRNA and other house-keeping genes using quantitative real-time PCR [71], [72], [73]. However, beta-actin is still widely used and is a reliable house-keeping gene in most expression studies in insects [74]. In this study, *beta-actin* was used as a reference gene in the evaluation of *AmVg* gene's expression. The expression of different house-keeping genes may change the dependence of stressors applied or other factors. Therefore, future gene expression studied in development or under stressors may require multiple housekeeping genes as reference genes.

It is well known that metals, such as Zn^2+^, can pass through the food chain. Our results showed that not only was predation by *H. axyridis* reduced on *A. medicaginis* exposed to high levels of Zn^2+^, but there was also a decrease in their ovipositioning rate (Figs. 6 and 7). A previously published study found that Mn and Zn were concentrated in mandible tips and were associated with increased hardness [75], however, it is unknown whether metals have negative effects when excess environmental metal is accumulated in insects. Although it is difficult to extrapolate these results higher up the food chain, it is likely that such accumulation higher up the food chain could also negatively impact on human health, perhaps even affecting human reproductive health. Thus, further studies to examine the biosafety and impact of metals on all elements of the biosphere are required.
